# Liquid Crystal Display-Based 3D Printing of Polylactic Acid/Microcrystalline Cellulose Composites

**DOI:** 10.3390/polym17243311

**Published:** 2025-12-15

**Authors:** Joyce Alves da Silva, Nayra Reis do Nascimento, Gilberto Garcia del Pino, José Luis Valin Rivera, Meylí Valin Fernández, Wanderson Veras da Silva, José Costa de Macedo Neto

**Affiliations:** 1 Programa de Pós-Graduação em Ciência e Engenharia de Materiais—PPGCEM, Universidade Federal do Amazonas (UFAM), Manaus 69067-005, Amazonas, Brazil; jads.engmateriais@gmail.com (J.A.d.S.); veras@ufam.edu.br (W.V.d.S.); 2 Conecthus Instituto de Tecnologia e Biotecnologia do Amazonas, Avenida Buriti, n°. 3001, Distrito Industrial I, Manaus 69075-000, Amazonas, Brazil; dra.nayrareis@gmail.com; 3 Departamento de Engenharia Mecânica, Universidade do Estado do Amazonas (UEA), Manaus 69050-020, Amazonas, Brazil; gpino@uea.edu.br; 4 Escuela de Ingeniería Mecánica, Pontificia Universidad Católica de Valparaíso, Valparaíso 2430000, Chile; 5 Department of Mechanical Engineering (DIM), Faculty of Engineering (FI), University of Concepción, Concepción 4070409, Chile; mvalin@udec.cl; 6 Departamento de Engenharia de Materiais, Universidade do Estado do Amazonas (UEA), Manaus 69050-020, Amazonas, Brazil

**Keywords:** microcrystalline cellulose, polylactic acid, 3D printing

## Abstract

This study explores the production of composites based on polylactic acid (PLA) reinforced with microcrystalline cellulose (MCC), using Additive Manufacturing technology via LCD. Polylactic acid, being biodegradable and possessing good mechanical properties, was combined with microcrystalline cellulose, which has a high modulus of elasticity, aiming to further improve its performance. Composites with different microcrystalline cellulose contents (1, 3, 5, and 10%) were obtained and compared to pure PLA. Characterization involved thermal, mechanical, morphological, and structural tests. The results showed that the addition of microcrystalline cellulose increases hardness, tensile strength, and modulus of elasticity. Scanning electron microscopy revealed more heterogeneous fracture surfaces in the composites compared to pure polylactic acid. Thermal stability varies according to the microcrystalline cellulose content, with increased degradation observed in some samples, reaching 1%. Increased water absorption was also detected with increasing microcrystalline cellulose concentration, indicating potential limitations in humid environments. The incorporation of microcrystalline cellulose, especially at moderate concentrations such as 3%, proved to be an effective strategy for improving the mechanical properties of polylactic acid.

## 1. Introduction

In recent years, various strategies have been studied with the objective of minimizing the pollution generated by materials of petrochemical origin and reducing the environmental impacts associated with their use in the application of these materials [1]. The polymers produced from raw materials of renewable sources are called biopolymers and have great potential as substitutes for polymers from fossil sources, such as in the case of polylactic acid (PLA) [2].

Polylactic acid (PLA) is a commercially available biodegradable polymer produced from renewable sources [3]. Due to the possibility of being produced from the bacterial fermentation of carbohydrates extracted from renewable sources such as millet, sugar cane, and sweet potatoes, it is considered a widely available biopolymer. It can be synthesized from the polymerization reaction of a monomer of natural origin (lactic acid), obtained by means of dextrose. It has the advantages of biocompatibility, not producing toxic or carcinogenic effects on most living beings. It is also bioabsorbent—a tiny polymer chain in vivo with subsequent elimination of material from the body’s metabolic pathways. Polylactic acid (PLA) is an ester-class polymer, obtained from renewable sources, produced from the polycondensation or polymerization of lactic acid by opening the milk ring [4,5].

As an alternative to improve the properties of biopolymers, the production of composites has been developed with the use of fillers that only come from renewable and biodegradable sources [6]. Microcrystalline cellulose (MCC) is obtained from natural cellulose and has different applications in various industrial sectors. Its applications can be found in general industries; however, MCC is mainly consolidated in applications of the food, pharmaceutical, and cosmetics industries. MCC is produced by the hydrolysis of cellulose cells, being extracted from the plant matter by means of a mineral acid that is diluted at its boiling temperature. The hydrolysis process removes most of the amorphous fraction and destroys the fibrillar morphology of cellulose, producing MCC [7].

Additive Manufacturing, through 3D printing, makes it possible to obtain complex structures on a small scale, from a variety of thermoplastic materials, including recycled ones [8]. Three-dimensional printing has become very popular in recent years due to the emergence of open-source projects and low-cost machines, making the technology accessible to all levels of users. At the same time, new materials are inserted into the market for application in this type of manufacturing technique, becoming increasingly necessary for the development of experimental material characterization studies to provide technical data to users [9].

The Additive Manufacturing (AM) process by photopolymerization is the principle of solidification of photopolymeric resin in a liquid state, layer by layer. This solidification occurs from ultraviolet (UV) or visible light. The incidence of light releases energy capable of initiating a chemical reaction in the liquid photopolymeric resin, hardening the material within the reservoir. Printers that use liquid photopolymers can be grouped into two main groups. Firstly, based on vector graphics, energy irradiation is directed specifically to a region of the layer to be cured, and secondly, based on the projection of masks or images, energy is directed over the entire length of a layer of the piece to be constructed [10].

The high-resolution photopolymerization of the Liquid Crystal Display (LCD) is included in the second group. Currently, photopolymerized printing accounts for 50% of the Additive Manufacturing market in the areas of mass reduction engineering, ultrasonic sensing, and functional constructions, as well as implementations in various fields of medicine, owing to its high capacity for obtaining fast and small printing speeds with great detail [11].

Studies in the literature have investigated PLA polymeric composites reinforced with microcrystalline cellulose (MCC) and natural fibers, with the main form of processing these composites being extrusion, followed by 3D printing using the Fused Deposition Modeling (FDM) technique. A patent by Moon et al. [12] demonstrates the use of cellulose and pyrocatechol in polymeric composites obtained by 3D printing via FDM, demonstrating the potential for reinforcement in Additive Manufacturing. At this time, there are no records of studies or patents that explore the incorporation of microcrystalline cellulose in PLA photopolymeric resin for 3D printing using the Liquid Crystal Display technique.

The novelty of this work lies in the use of Additive Manufacturing through the LCD technique to obtain polylactic acid composites with microcrystalline cellulose. Since the addition of natural fibers to PLA has not been studied, few studies investigate the influence of the process using the LCD technique in obtaining the composite material, which may directly impact the homogeneity of charge dispersion, interfacial adhesion, and the final performance of the material.

Furthermore, the research seeks to determine the optimal proportion of microcrystalline cellulose in polylactic acid to evaluate how different concentrations affect thermal degradation, mechanical resistance, and water absorption of the material. This knowledge can contribute to the development of more sustainable and efficient biocomposites, expanding their applications in the industry and reducing the environmental impact of two conventional materials.

## 2. Materials and Methods

### 2.1. Materials Used

The commercial resin Esun PLA Bio UV, a pre-pigmentation photocured, (Esun 3D, Shenzhen, China) was used for this study with the following properties according to the manufacturer: Shore Hardness of 78–80; Viscosity from 200 to 300 M Pa.s; density of 1.09–1.10 g/cm^3^; Traction resistance of 37–48 MPa; Breaking resistance of 28–28% and Impact Resistance (Izod) of 32 to 36 J/m. The microcrystalline Cellulose used was supplied by ACS scientifically and has a Polymerization Grade Máx. 350; pH 5.0–7.5; heavy metals Max 0.001%; Ignition residues Max. 0.1%; density of 0.26–0.33 g/cm^3^.

To obtain the mixture of polylactic acid and MCC, mass variations of 1, 3, 5, and 10% of MCC, in relation to polylactic acid, are needed. To measure these masses, an analytical balance (L Series LA110, ACCULAB, New York, NY, USA) was used. The mixtures were carried out using a magnetic stirrer (752 A, FISATOM, São Paulo, Brazil) for 20 min, at a standard temperature of 20 °C to 25 °C.

### 2.2. Additive Manufacturing of Test Specimens

Firstly, the printing of the test specimens was planned, and the samples were designed using Solidworks 2023 software and subsequently converted to STL (Standard Tesselation Language) format. In addition to the printing process, for this purpose, the free version of the Chitubox^®^ software, version 1.9.3, was compiled, in which the printing parameters, such as exposure time, speed, layer height resolution, and other essential functions for the process, were defined. Photopolymerization was performed in a tank attached to the printer. We opted to use the fatigue parameters indicated by the manufacturer in order to achieve the mechanical properties provided in the technical data. We also decided to print the test specimens horizontally, with a larger area in contact with the surface of the platform and without support, due to the effects of anisotropy and the inclusion of supports mentioned above in this work. The parameters used are described in Table 1.

For printing the test specimens, the Elegoo Mars 2 Pro 3D printer (Elegoo, Shenzhen, China) was used. This printer has a monochromatic LCD type, which makes it possible to significantly reduce the curing time between layers, making the printing process faster, with a print volume of 129 × 80 × 160 mm, precision of 50 μm, and Z axes, and perform 1 to 2 s of cure per layer.

Before the printing process of the samples, perform the following steps:

(1) Verification of the level of the printing platform;
(2) Certification of tension of FEP (Fluorinated Ethylene Propylene—transparent polymer film resistant to heat, used at the bottom of the resin tank in 3D printers).

For the test specimens of the traction forms, the dimensions of type V of the ASTM D638 (2017) [13] standard were used. These dimensions are only recommended for 30 materials of different stiffness values, generally used in comparative studies. A total of 30 type V test specimens were manufactured, 5 for each approved condition (0%, 1%, 3%, 5% and 10% MCC) for the water absorption test, in accordance with the standard requirements.

After printing, the approved samples are attached to the Mercury Plus 2 Elegoo Wash & Cure brand wash and cure machine (Elegoo, Shenzhen, China). The washing function is conducted in 95% isopropyl alcohol for 3 min. And immediately, they were subjected to post-cure within a period of 15 min.

### 2.3. Characterization of Microcrystalline Cellulose

#### 2.3.1. Scanning Electron Microscopy with X-Ray Energy Dispersive Spectroscopy

The morphology of the samples was carried out with the scanning electron microscopy (SEM) equipment, the JEOL model IT500HR (JEOL, Tokyo, Japan), with the objective of identifying the microparticles and the morphology of the material. X-ray energy-dispersive spectroscopy (EDS) analyses are only used for qualitative and semiquantitative microanalysis of chemical elements present in non-materials. The samples are coated with gold metallization, with a thickness of 15 to 20 nm, and the voltage used in the equipment is 5 kV.

#### 2.3.2. Fourier-Transform Infrared Spectroscopy

The objective of the analysis was to identify the functional groups present in the microcellulose. In the experimental procedure, an Agilent Technologies infrared spectrometer was used, model Cary 630 (Agilent, Santa Clara, CA, USA), with a nominal resolution of 8 cm^−1^, in a spectral range from 4000 to 650 cm^−1^, in transmission mode, with 128 variations. The data are obtained using Origin 2016 software. The samples analyzed are in the form of a manual.

#### 2.3.3. X-Ray Diffraction

X-Ray Diffraction (XRD) analysis of microcrystalline cellulose was carried out through the following method. The test was carried out with an analytical brand diffractometer, model X’PERT PRO MRD (Malvern Panalytical, Malvern, UK). The operating conditions are found in Table 2.

The calculation of the Crystallinity Index (Ic) of the MC samples was carried out according to the amorphous halo subtraction method. Through this method, a baseline adjustment was applied to the spectrum of the amorphous material using the Origin Pro 8.0 software. In this way, after the substrate of the amorphous, or vitreous, spectrum, from the original spectrum, none of the resulting spectra contain a negative signal. Assim, or Ic, is calculated by the ratio between the area of the crystalline region and the total area, according to Equation (1):

(1) $$\mathrm{IC}\;\%\;=\;\frac{Ac}{At}\;\times \;100$$

where At is the total area of the XRD curve, and Ac is the sum of the areas of two peaks that correspond to the crystalline structure of cellulose, calculated after correction of the baseline. For the calculation of areas, the remaining peaks in the DRX curves are disregarded. The crystallographic planes were analyzed and identified by means of the Crystallographic Information File (CIF), through the Vesta 4.6.0 software, which offers the crystallographic information essential to determine the crystal structure.

### 2.4. Characterization of Polylactic Acid and Composites

#### 2.4.1. Scanning Electron Microscopy

The morphology of the samples was carried out with the scanning electron microscopy (SEM) equipment of the JEOL brand model IT500HR, with the objective of identifying the microparticles, at the intersection of the deposits of the layers, the morphology of the material and the characteristic of the fracture of the samples to the dispersion of the non-composite micro-cellulose particles before and after or thermal aging. The samples are coated with gold metallization, with a thickness of 15 to 20 nm, and the voltage used in the equipment is 5 kV. The MEV analysis was carried out after the training test.

#### 2.4.2. Fourier-Transform Infrared Spectroscopy

To obtain two spectra in the infrared region with Fourier-Transform (FTIR) for polylactic acid and composite materials (PLA + MCC), the Agilent Technologies brand infrared spectrometer was used, model Cary 630, with a nominal resolution of 8 cm^−1^, in a spectral range from 4000 to 650 cm^−1^, no transmission mode, with 128 sweeps. The data are obtained using Origin 2016 software.

#### 2.4.3. Thermal Analysis by Differential Scanning Calorimetry and Thermogravimetric Analysis

Differential Scanning Calorimetry (DSC) techniques are used for glass transition temperature (Tg) characterization, and Thermogravimetric Analysis (TGA) for mass loss analysis. Knowing the thermal properties of both pure and composite polylactic acid is of utmost importance in evaluating the processing conditions and applicability of the same conditions that require thermal resistance. The TGA/DTG curves were obtained with a temperature rate of 10 °C/min at a final temperature of 800 °C, both conducted under a nitrogen atmosphere (flow rate of 30 mL/min), with a flow rate of 10 °C/min. The test was carried out on the TA Instruments SDT Q600 (TA Instruments, New Castle, DE, USA) simultaneous thermal analyzer.

#### 2.4.4. X-Ray Diffraction

The crystalline structure of samples of pure polylactic acid and two composites was evaluated using the X-Ray Diffraction (XRD) technique. The test was carried out with an analytical brand diffractometer, model X’PERT PRO MRD. The operating conditions of the analysis are found in Table 3.

#### 2.4.5. Tensile Test

To carry out the tensile test, a mechanical testing machine (5583, INSTRON, Norwood, MA, USA) was used, as well as a load cell of 1.0 kN, resolution of 0.01 kg.f, and test speed of 0.5 mm/min. The test was carried out in accordance with ASTM-D638, in a room with controlled temperatures of 23 ± 2 °C and relative humidity of 50 ± 5%, as well as a stabilization time of these conditions greater than 3 h. Five test specimens for each composition (0, 1, 3, 5, and 10% MCC) before thermal aging were tested.

#### 2.4.6. Shore D Hardness

The hardness test was carried out at room temperature, using the Shore type D hardness tester, model Dp-100, from the Instrutherm brand (Sao Paulo, Brazil). Five indentations were carried out for each condition and on one of the faces of the samples, calculating an average value of the hardness. The test procedure was applied according to the ISO 868:2003 standard [14].

#### 2.4.7. Water Absorption Test in Composites

The water absorption tests of two raw materials were carried out in accordance with the ASTM D 570-98 standard [15]. The test specimens were measured, weighed, and immersed in distilled water at room temperature. At pre-determined time intervals, the samples are removed from the water, dried with a paper towel, weighed on a precision scale (±0.1 mg), and replaced again in the water.

This procedure was repeated for approximately 12 weeks, when the point of saturation of the materials in relation to water absorption was reached. At the end of the test, a curve of water absorption (%) versus immersion time (days) was obtained, in accordance with the standard. The percentage of water absorption in composites and polymers will be calculated from Equation (2):

(2) $$\Delta Ma\;(\%)\;=\;\frac{Mf-Mi}{Mi}\;\times \;100$$

where Δ*Ma* is the variation in water absorption, and Mi and Mf correspond to the masses shown before and after immersion in water.

## 3. Results

### 3.1. Obtaining Composites

**Figure 1:**
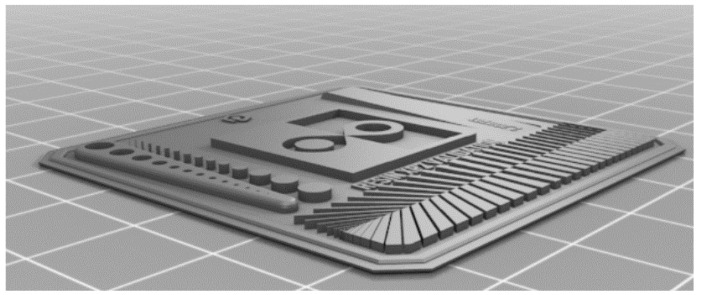
Validation model for 3D printing.

Process parameter definitions are crucial for Additive Manufacturing. The first step in parameterizing a resin is a validation model. This model includes several different details of various sizes and is used to assess the quality and dimensional accuracy of the print, as illustrated in Figure 1.

The printing parameters were based on the 3D cure printing process and the recommended predefined parameters in the Chitubox slicer for the Elegoo Mars 2 Pro printer. Fine adjustments can be made later based on the results obtained from this print.

During processing, defects were observed, such as gaps, layer misalignment in the test specimen due to an uneven platform, loose fluorinated ethylene propylene (FEP), and the presence of solid residue in the tank.

**Figure 2:**
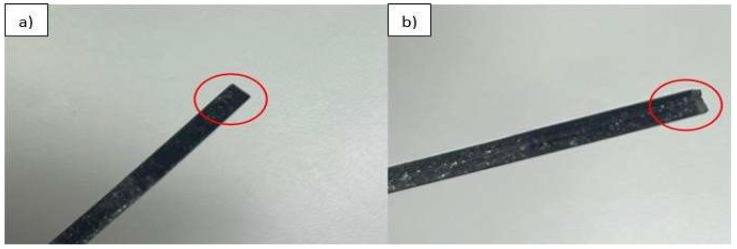
(**a**) Aligned layers. (**b**) Misalignment between layers.

Figure 2b shows the layer misalignment issue. To resolve this issue, it was necessary to recalibrate the printer to ensure correct alignment between the LCD screen and the platform. The FEP was kept taut to ensure the layers were always perfectly aligned, as shown in Figure 2a.

**Figure 3:**
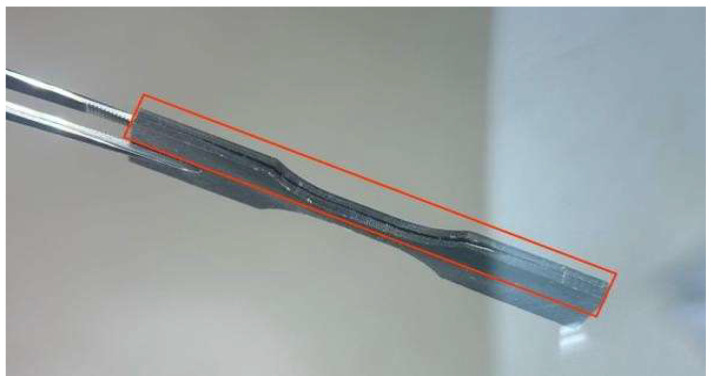
Delamination failure between layers.

As shown in Figure 3, delamination between layers was also observed in the polylactic acid reinforced with 10% microcrystalline cellulose, probably related to the excessive presence of solid residues in the tank. For this, it was necessary to stir the resin together with the microcrystalline cellulose that was in the tank before printing; the stirring was performed manually.

### 3.2. Vibrational Fourier-Transform Infrared Absorption Spectroscopy

**Figure 4:**
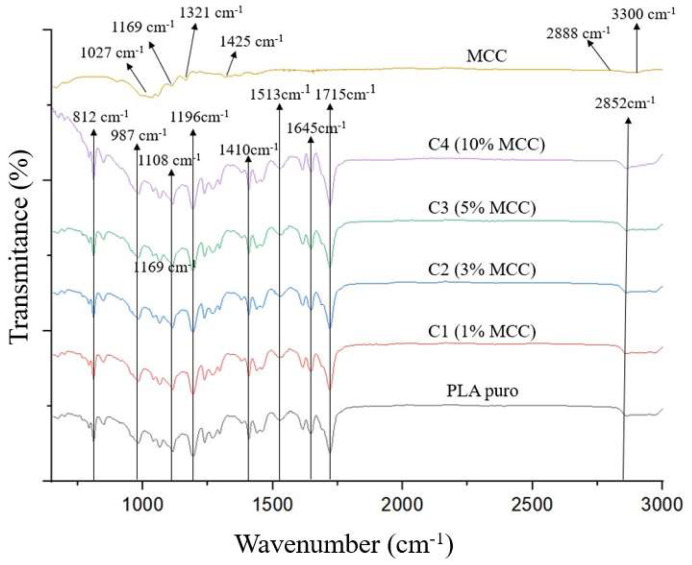
Infrared absorption vibrational spectrum (FTIR) obtained for microcrystalline cellulose, pure PLA, and composites (PLA + MCC).

Figure 4 shows the FTIR spectra of the functional groups for the microcrystalline cellulose sample, pure polylactic acid, and the composites (PLA + MCC). The first peak identified was the 1027 cm^−1^ band for the microcrystalline cellulose spectrum, attributed to C-O stretching, possibly due to C-O vibration forces and the movement of C-H vibrations present in the cellulose [16]. Other cellulose and hemicellulose bands appeared around 1169 cm^−1^ and 1321 cm^−1^, referring, respectively, to CH2 agitation and valence vibration of C–O bonds [17]. Another peak observed in the sample around 1428 cm^−1^ is due to aromatic skeletal vibrations combined with C-H in-plane deformation [18].

The peaks observed in the wavenumber range of 3300–2888 cm^−1^ are characteristic of the stretching vibration of OH and C-H bonds in polysaccharides [19]. The broad peak at 3330 cm^−1^ is characteristic of the stretching vibration of the hydroxyl group in polysaccharides [20]. This peak also includes vibrations of intermolecular and intramolecular hydrogen bonds in cellulose. The band at 2888 cm^−1^ is attributed to the C-H stretching vibration of all hydrocarbon constituents in polysaccharides [21].

These bands identified in the microcellulose sample are similar to information reported in the literature, such as in the work conducted by Suryanegara and his team [22], who also observed the same peaks in their samples, confirming that the product used does indeed correspond to cellulose.

Table 4 and Figure 4 show the FTIR spectra obtained for pure polylactic acid and PLA/MCC composites at different concentrations. Peaks corresponding to the crystalline and amorphous phases of polylactic acid were identified, demonstrated by the bands present at wavenumbers of 812 cm^−1^ (due to C=O bond deformation) and 987 cm^−1^ (due to C-COO stretching), respectively [23].

The symmetric stretching of COC is present in two peaks, located at 1108 and 1196 cm^−1^, which were also observed by Silva [24]. The bands at 1410, 1513, and 1645 cm^−1^ are attributed to the CH(CH3) vibration, and the peak present in the region of 1715 cm^−1^ refers to the stretching of the C=O ester group, which can confirm the presence of the methacrylate group. Bands were observed at the wavenumber around 2852 cm^−1^, characterizing PLA due to the presence of the C-CH3 group. The vibration corresponding to CH(CH3) appears at the wavenumber close to 1410 cm^−1^. Furthermore, a band related to the C=O stretching was identified at approximately 1196 cm^−1^, also associated with the PLA structure [25].

FTIR analysis performed on composites with different microcellulose concentrations (0, 1%, 3%, 5%, and 10% MCC) showed similar functional groups, with peaks located in the same wavelength ranges.

Comparing the samples, it was concluded that no new peaks emerged, nor did existing peaks diminish as a result of polylactic acid processing. This same result was obtained by Bartolomei [23] when processing polylactic acid with the addition of crystalline nanocellulose. This indicates that no significant changes occurred in the chemical structure or functional bonds of the sample. The absence of changes in the spectrum suggests that there were no strong chemical interactions, such as the formation of new covalent bonds, but only physical interactions, such as Van der Waals forces. Similar results were observed by Matos [26], who, when processing nanocellulose with polylactic acid, found that there was no emergence of new peaks nor diminishment of existing peaks.

### 3.3. Mechanical Tests

#### 3.3.1. Tensile Test

The mechanical tensile strength results of the printed specimens are shown in Table 5. The average tensile strength values using polylactic acid as a reference show a significant increase in this property in the composites with microcrystalline cellulose. Sample C2 (3% MCC) stood out as the best performer, presenting a 23.80% increase in average tensile stress compared to pure polylactic acid. On the other hand, sample C4 (10.00% MCC) presented the smallest increase, with an increase of only 4.10%. For the intermediate samples, C1 (1% MCC) and C3 (5% MCC), the increases in tensile stress were 4.30% and 11.33%, respectively, when compared to pure polylactic acid.

The inferior performance of sample C4 may be associated with inadequate dispersion of crystalline microcellulose (MCC) in the polylactic acid matrix. This result may occur due to the formation of microcellulose agglomerates that are not uniformly dispersed throughout the polymer matrix [27]. These agglomerates can act as stress concentration points, thus compromising the composite’s tensile strength. According to Santos [28], inadequate dispersion of crystalline microcellulose reduces the interaction between the matrix and the reinforcement, negatively impacting the material’s mechanical performance.

Table 6 shows the average values of the elastic modulus of pure polylactic acid and the composites. The data show a progressive increase in the elastic modulus of composites C1 (1.00%), C2 (3.00%), C3 (5.00%), and C4 (10.00%) compared to pure polylactic acid. The largest increase was recorded in sample C4 (10.00%), with an elastic modulus 38.53% higher than polylactic acid. Sample C2 (3.00%) also stood out, with an increase of 32.11% compared to pure polylactic acid. For samples C1 (1% MCC) and C3 (5% MCC), the increases were 6.88% and 27.98%, respectively.

This increase in stiffness can be explained by the very nature of microcrystalline cellulose. It has a highly ordered crystalline structure, resulting in a high modulus of elasticity. When MCC is incorporated into polylactic acid, it acts as a mechanical reinforcement, transferring part of the stresses applied to the material to its fibers. This occurs because microcellulose fibers, being stiffer than the polymer matrix, support a greater portion of the load, reducing deformation of the PLA matrix and, consequently, increasing the overall stiffness of the composite [26,29].

**Figure 5:**
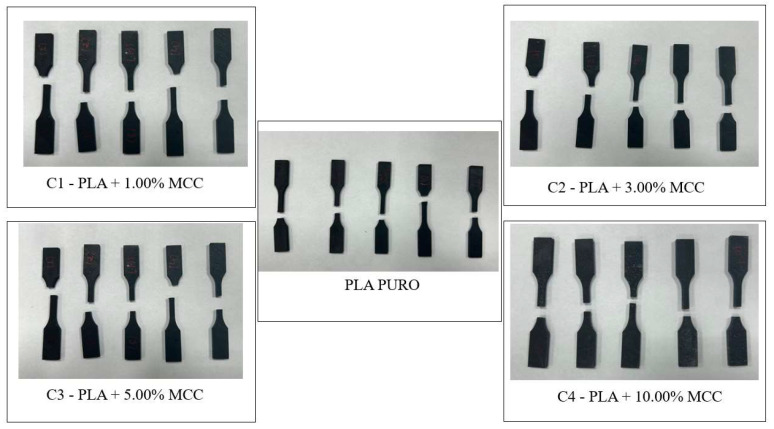
Test specimens after rupture in the tensile test.

**Figure 6:**
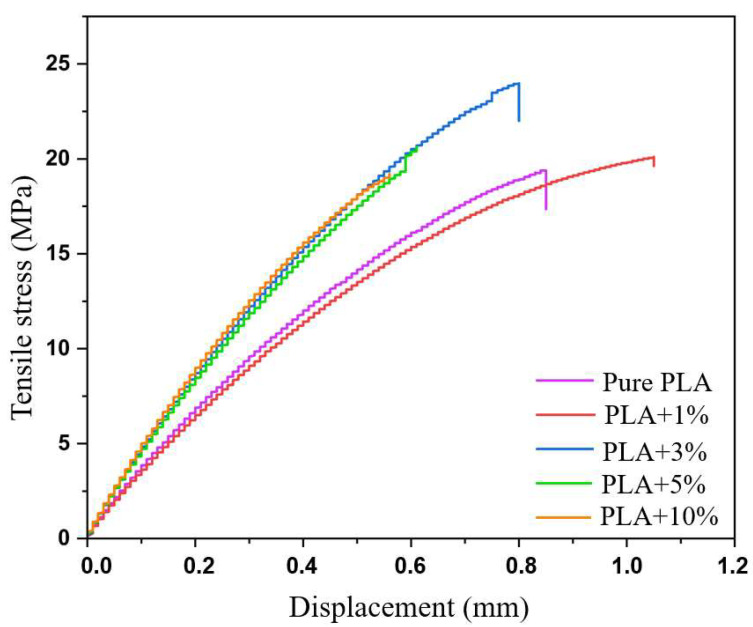
Stress–strain curves of polylactic acid and composites (PLA + MCC).

Figure 5 shows the composite specimens (PLA + MCC) after fracture in the tensile test. It can be observed that, although in some cases, the fracture occurred outside the central region, most specimens fractured approximately in the middle of the gauge length, exhibiting low elongation. This behavior is consistent with the results of the stress–strain curve shown in Figure 6, in which all compositions show little deformation until fracture. This mechanical response is associated with the intrinsically rigid and relatively brittle nature of polylactic acid, which limits the ductility of the material. The addition of crystalline microcellulose reinforces this effect, further increasing the stiffness of the composite and contributing to fracture with reduced elongation [30].

#### 3.3.2. Shore Hardness

The results obtained in the Shore D hardness test for pure PLA and for the composites at different concentrations are shown in Table 7. It can be seen that the hardness value increases as the MCC loading concentration also increases. Sample C1 with the addition of 1% microcrystalline cellulose (MCC) showed a 6.75% increase in hardness compared to pure PLA. This is significant, especially considering such a small amount of additive. For samples C2 (3% MCC), C3 (5% MCC), and C4 (10% MCC), the increases in hardness compared to PLA without the addition of microcrystalline cellulose were 7.39%, 10.66%, and 15.57%, respectively.

Increasing the percentage of MCC generates a continuous increase in the hardness of the material. This occurs because MCC, being a crystalline and rigid material, improves the strength of the PLA matrix [22]. The maximum hardness was observed in sample C4, with 10% MCC, suggesting that larger amounts of MCC continue to reinforce the material; therefore, MCC acts as a reinforcement in the polylactic acid polymer matrix, better distributing the applied surface stresses [29].

### 3.4. X-Ray Diffraction

**Figure 7:**
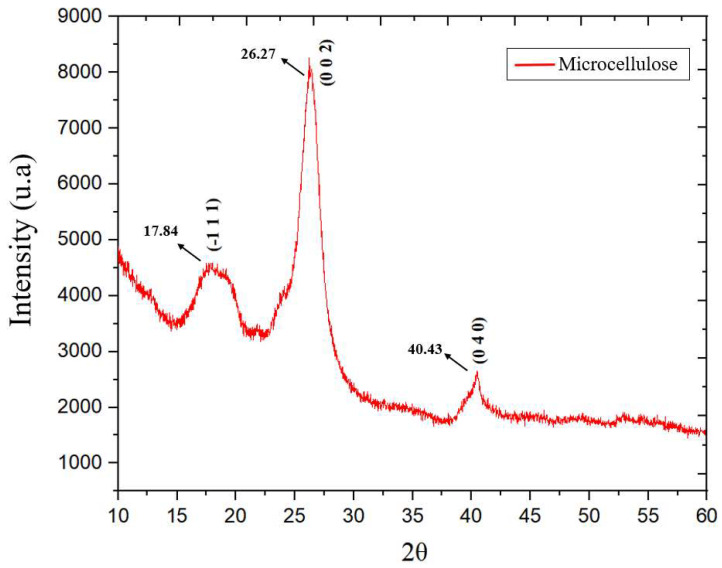
Microcrystalline cellulose XRD Curves.

**Figure 8:**
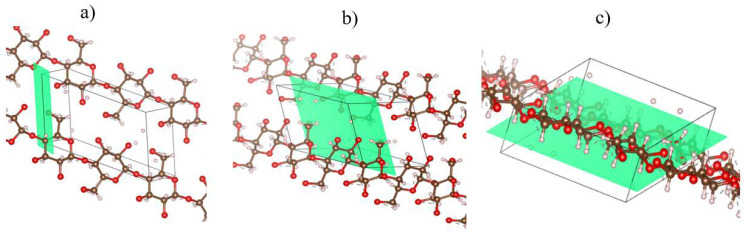
Crystallographic planes: (**a**) plane (−1 0 1); 2θ = 17.84°; (**b**) plane (0 0 2); 2θ = 26.27°; (**c**) plane (0 4 0); 2θ = 40.43°. Red colors indicate the molecules, while green represents the crystallographic plane.

The XRD curve obtained from the analysis of microcrystalline cellulose (MCC) is shown in Figure 7. Figure 8 shows the planes traced in the monoclinic unit cell attributed to the crystal structure of MCC. These aspects were analyzed and identified using the Crystallographic Information File (CIF) in Vesta software, which provides essential crystallographic information for determining the crystal structure.

The XRD curves show the characteristic peaks of the crystal structure of type I cellulose [31]. The peak of greatest intensity was observed around 2θ = 26.27°, corresponding to the (0 0 2) plane (Figure 8b). Furthermore, a less pronounced peak was identified at 2θ = 17.84°, corresponding to the (−1 0 1) plane (Figure 8a), and a well-defined peak at 2θ = 40.43°, associated with the (0 4 0) plane (Figure 8c) [32].

Similar results were identified by Jesus [33] when studying cellulose microfibrils (CMF) extracted from curauá fiber, where main peaks were recorded at 2θ = 22.9° and 2θ = 15.2°. Similarly, Mendes [21] in his research observed X-Ray Diffraction peaks from microcrystalline cellulose (MCC), finding typical reflections associated with the crystalline phases of cellulose at 2θ = 17.06°, 2θ = 26.22°, and 2θ = 40.26°.

**Figure 9:**
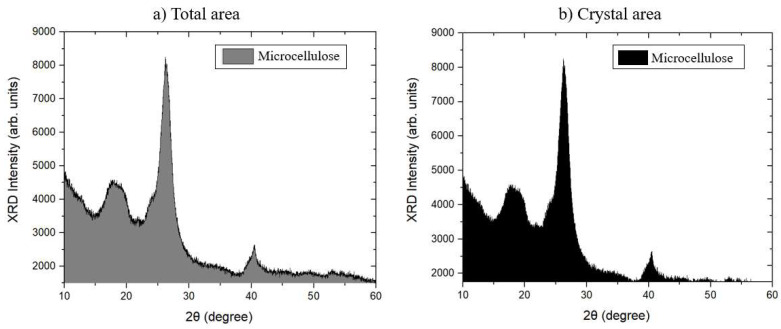
Model of curves used to calculate the Crystallinity Index (Ic).

From the curves obtained, it was possible to calculate the crystallinity index (Ic) by subtracting the amorphous halo. Figure 9 illustrates the modification of the baseline of the curve and the areas used to calculate the Ic.

Table 8 shows the crystallinity indices calculated from X-Ray Diffraction (XRD) curves using Equation (1). The results indicate that the Crystallinity Index (Ic) of the analyzed MCC was 40.54%, suggesting a balance between crystalline and amorphous regions present in the structure of crystalline microcellulose.

Similar results were obtained by Mendes (2023) [21], who found an Ic of 44.90% for crystalline microcellulose (MCC), demonstrating that crystallinity can vary slightly depending on the extraction and purification method adopted.

**Figure 10:**
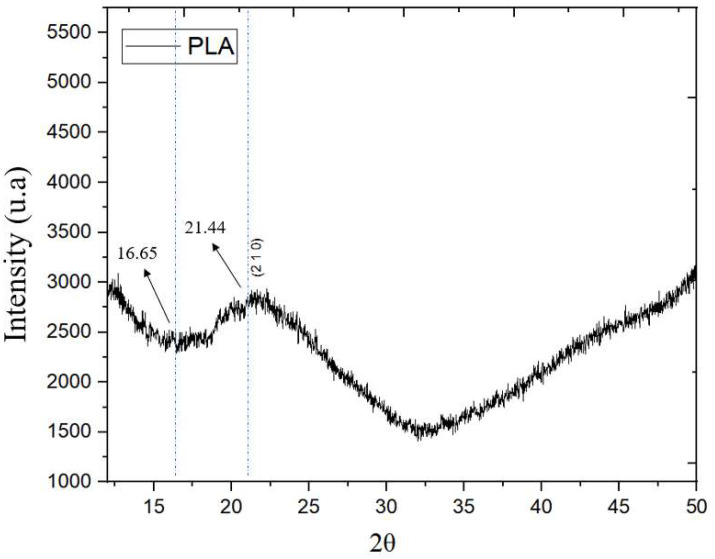
XRD diagram of pure polylactic acid. Dotted line indicates the peak position.

**Figure 11:**
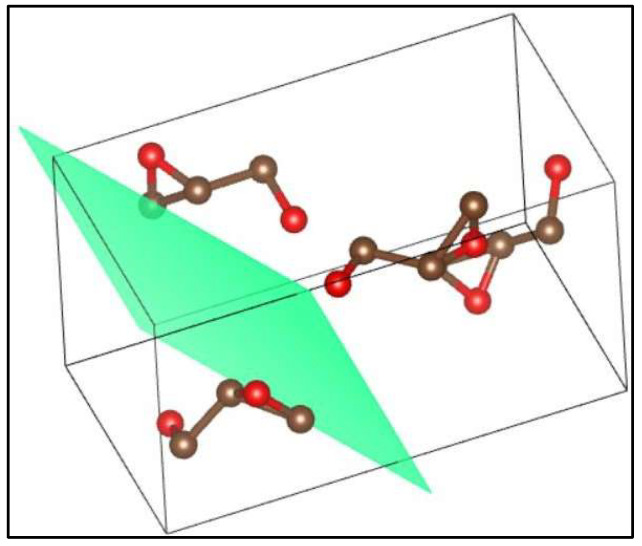
Plane (2 1 0); 2θ = 21.44°. Red colors indicate the molecules, while green represents the crystallographic plane.

Figure 10 shows the curves obtained by XRD for pure polylactic acid, whose semicrystalline structure is associated with the orthorhombic system. Two characteristic peaks of pure polylactic acid are detected at 2θ = 16.65° and 21.44°, indicating the semicrystalline state of the material. The most intense peak is 2θ = 21.44°, referring to the (2 1 0) plane (Figure 11); the more intense the peak, the more considerable the crystallinity of the material [34].

In the work of Matos [26], similar peaks were also found in a wire produced only with polylactic acid. Three peaks were observed: 2θ = 16.7°, 2θ = 30.9°, 2θ = 32.7°. The other wires presented only the peak around 2θ = 16.0°, which corresponds to the α phase of the polylactic acid crystal.

**Figure 12:**
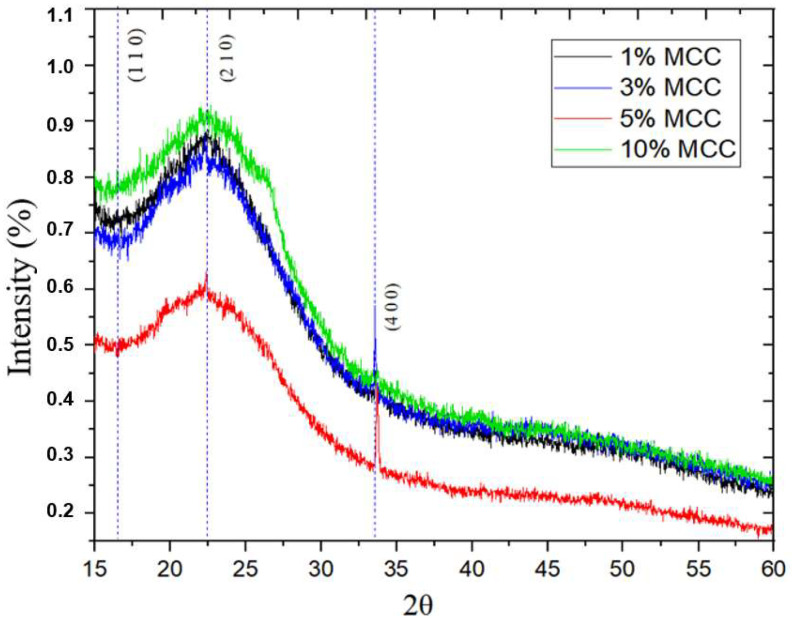
XRD diffractogram of composites at different MCC concentrations.

Figure 12 shows the curves obtained by XRD for the composite materials at different microcrystalline cellulose concentrations (1%, 3%, 5%, and 10% MCC). It can be noted that some peaks remained similar to those obtained for pure polylactic acid. Three peaks were observed: 2θ = 16.56°, 2θ = 22.58°, and the peak 2θ = 33.56° that appeared for the composite materials of 1%, 3%, 5%, and 10% MCC.

However, the intensification of the peak at 2θ = 33.56° in the samples with MCC may be related to a contribution from the microcellulose itself, which has a distinct crystalline structure from that of polylactic acid. In the study by Matos [26], a peak was identified in the diffractogram at 2θ = 32.7° for polylactic acid composites with microfibrillated cellulose, a value very close to the 33.56° peak observed in the polylactic acid composites with microcrystalline cellulose (MCC) in this study. This peak may be related to the intensification of the material’s crystallinity or the formation of a new crystalline phase. This data reinforces the hypothesis that microcellulose acts as a nucleating agent, promoting greater organization in the PLA polymer matrix, especially at higher MCC concentrations [28]. However, an increase in the crystalline fraction does not necessarily translate into greater toughness. Higher crystallinity, combined with the excavating nature of MCC, tends to reduce the deformation capacity of the matrix, which explains the low elongation observed and the greater brittleness at high concentrations. This behavior is aggravated by the formation of agglomerates and voids, as evidenced by SEM, which act as stress concentrators and favor premature fracture.

Furthermore, Zhong [35] and his team conducted studies on the crystal structure of pure polylactic acid, identifying peaks in the diffractogram that were attributed to β-phase crystals. They observed a peak at 2θ = 16°, characteristic of the amorphous phase of polylactic acid, as well as peaks near 26° and 33°, which are typically associated with the β-phase crystalline phase of polylactic acid. These results are relevant because they suggest that polylactic acid has different types of crystals depending on its structure and processing conditions.

Based on this, it can be hypothesized that, with the addition of microcrystalline cellulose, a change in the formation or proportion of these crystalline phases of polylactic acid, including the β-phase, may occur. Microcrystalline cellulose may directly interfere with the growth and arrangement of polylactic acid polymer chains, favoring the development of crystals in certain orientations, which would explain the intensification of the peak at 33.56°.

### 3.5. Microcrystalline Cellulose Scanning Electron Microscopy

**Figure 13:**
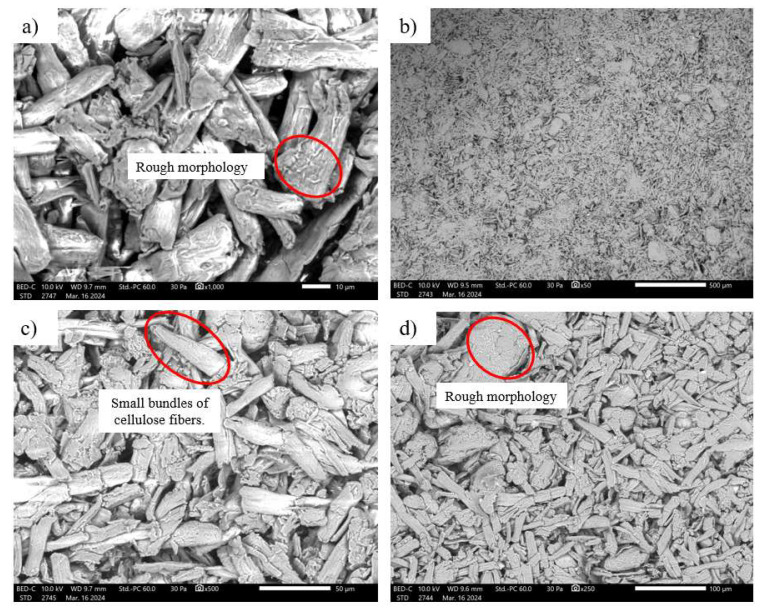
SEM of MCC powder: (**a**) MCC powder at 1000× photographic magnification; (**b**) MCC powder at 50× photographic magnification; (**c**) MCC powder at 500× photographic magnification; (**d**) MCC powder at 250× photographic magnification.

Figure 13 shows the micrographs of microcrystalline cellulose powder obtained through scanning electron microscopy (SEM). In the observations of the SEM images, an irregular and rough morphological structure that microcrystalline cellulose presents in its microcrystalline and powder form is notable. According to Santos and Tavares [28], microcrystalline cellulose presents a wide variety of sizes and shapes, most of them with an elongated profile.

Higher magnification of the images reveals that many of these particles are composed of small bundles of cellulose fibers. This occurs because microcrystalline cellulose particles tend to self-aggregate due to the presence of hydroxyl groups on their surfaces [28].

Note that microcrystalline cellulose has a rough surface, likely due to the removal of lignin, hemicellulose, and other impurities that surrounded and held the fibrils together, removed during the acid hydrolysis process [32]. MCC is derived from high-quality wood pulp by acid hydrolysis, so that once the amorphous regions are removed, cellulose crystal aggregates can form [7]. However, it is generally observed that microcrystalline cellulose has an irregular shape, allowing for a variety of shapes and characteristics within a single sample.

### 3.6. Fractography of Pure Polylactic Acid and Composites (PLA + MCC)

**Figure 14:**
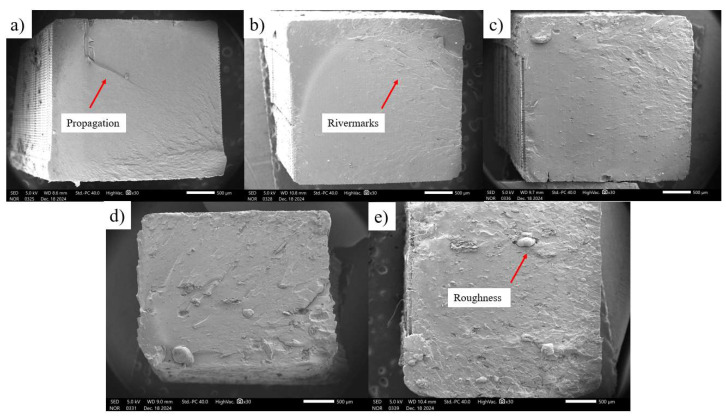
Morphology of the fracture region of the specimens of the samples: (**a**) pure PLA; (**b**) C1 (1% MCC); (**c**) C2 (3% MCC); (**d**) C3 (5% MCC); (**e**) C4 (10% MCC) (30× magnification).

SEM analysis was performed on the fracture morphology of the specimens after tensile testing. The micrographs obtained (Figure 14) show the pure PLA matrix and the composites containing microcrystalline cellulose (MCC) at concentrations of 1%, 3%, 5%, and 10%. Figure 14 shows the dispersion of microcrystalline cellulose (MCC) in the PLA polymer matrix. It is noteworthy that, as the microcrystalline cellulose concentration in the polylactic acid matrix increases, the roughness of the fracture surface increases. This behavior was expected due to the formation of agglomerates with increasing microcrystalline cellulose percentage. Studies in the literature also indicate that increasing microcrystalline cellulose in the polylactic acid matrix intensifies agglomeration at the interface between both components, which can impact interfacial adhesion and the mechanical properties of the material [26,36,37].

Figure 14a clearly demonstrates crack propagation. This crack tends to propagate in a brittle mode, especially in regions of lower cohesion between the deposited layers. Final shear failure occurs simultaneously with the coalescence of the crack lines. Figure 14b shows the so-called river marks, typical of multi-plane crack propagation, which occur along the grain direction and often present a bifurcation pattern similar to “feathers.” All samples clearly illustrate the transition region from the fracture initiation, a smoother region, to the rapid fracture region, a rougher region [38].

Figure 14c shows a surface with more uniform crack propagation, but “river marks” were also evident, while Figure 14d,e show roughness and irregularities, indicating failure regions associated with the presence of pores, poorly dispersed particles, or residual stresses. The roughness contributes to increased energy dissipation during fracture. The fracture morphology, as observed in these images, confirms that the failure mechanisms in PLA/MCC composites are complex and multifactorial, involving everything from crack nucleation to their propagation through different modes, varying according to the load concentration and the quality of the matrix/reinforcement interface [39].

**Figure 15:**
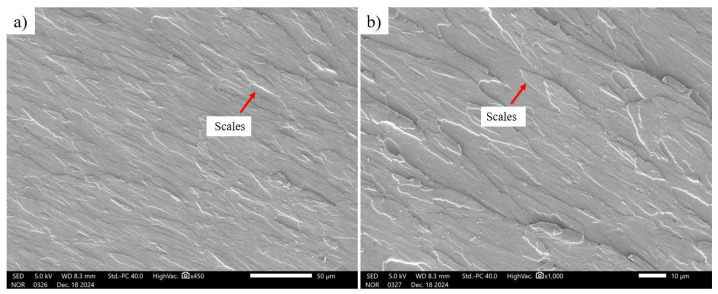
Morphology of the fracture region of the pure polylactic acid specimens: (**a**) 450× (**b**) 1000×.

In Figure 15, it can be observed that the fracture of pure polylactic acid presented an overlapping layer surface on the fracture surface, which exhibits a brittle fracture appearance, without apparent plastic deformation (Figure 15a), and this characteristic is maintained even at higher magnification (Figure 15b). This behavior was also observed by Santos (2020) [40], who found a “scale-like” morphology for pure polylactic acid, confirming the brittle characteristic of this polymer. Silva [24] also observed the brittle fracture of pure polylactic acid, evidenced by the presence of overlapping layers on the fracture surface, without the occurrence of plastic deformation.

**Figure 16:**
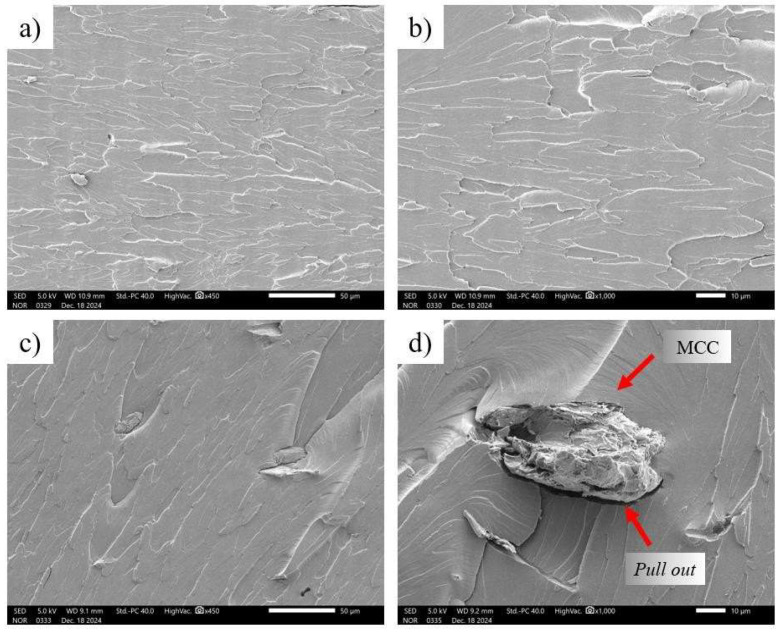
Morphology of the fracture region of the specimens: (**a**) C1 (1% MCC) 450×, (**b**) C1 (1% MCC) 1000×, (**c**) C2 (3% MCC) 450×, and (**d**) C2 (3% MCC) 1000×.

In composites containing microcrystalline cellulose, the fracture roughness varied with the reinforcement content. Figure 16a,b show that the surface of sample C1 (1% MCC) remained relatively homogeneous, indicating that the low amount of filler did not significantly alter the fracture morphology. This behavior was also reported by Krapez et al. [36], who analyzed the addition of 1% microcrystalline cellulose in wood and polylactic acid filaments, where microscopic observations showed that MCC was well distributed in the matrix. Furthermore, it is well established that for cellulose micro- and nanoparticles to effectively act as reinforcement in composite materials, they must be uniformly dispersed in the polymer matrix. This uniform dispersion is essential because it prevents the creation of substantial stress concentrations in the polymer matrix [41].

In Figure 16c,d, the roughness of sample C2 (3% MCC) was slightly higher compared to pure polylactic acid, and in sample C1 (1% MCC), a void was also observed between the microcrystalline cellulose and the polylactic acid matrix, indicating little adhesion between the microcrystalline cellulose and polylactic acid, although homogeneous fracture still predominated. This concentration showed the best performance in the tensile test, suggesting that 3% microcrystalline cellulose is a balance point that improves mechanical strength without compromising structural uniformity. This behavior is similar to that reported by Santos et al. [40], who observed that low coconut fiber contents in polylactic acid biocomposites resulted in more uniform fractures due to the good dispersion of fillers in the matrix.

**Figure 17:**
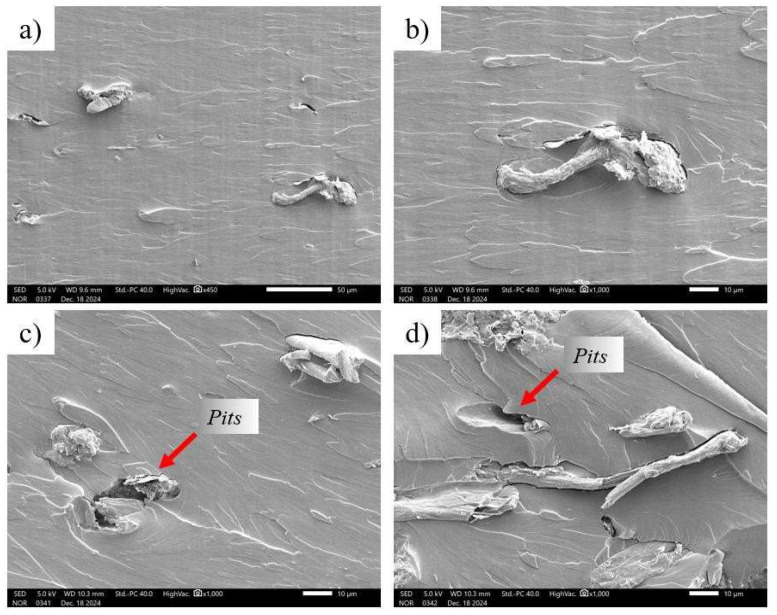
Morphology of the fracture region of the test specimens of the samples (**a**) C3 (5% MCC) 450×, (**b**) C3 (5% MCC) 1000×, (**c**) C4 (10% MCC) 450×, and (**d**) C4 (10% MCC) 1000×.

In Figure 17a,b, corresponding to sample C3 (5% MCC), heterogeneous regions with evidence of filler aggregation were observed, in addition to the presence of voids in the polylactic acid matrix, known as pits. These voids suggest a deficient interfacial interaction between the microcrystalline cellulose and the matrix, indicating that part of the filler was detached during fracture. This effect was even more evident in sample C4 (10% MCC), as shown in Figure 17c,d, in which the presence of accentuated agglomerates resulted in interfacial failures and increased fracture surface roughness. Similar behavior was reported by Silva [24] when analyzing polylactic acid composites with bamboo-sandpaper fiber, in which most of the fibers broke along with the matrix, while others were torn off during the tensile test, as evidenced by the presence of holes in the fracture surface. Furthermore, a gap was observed at the fiber–polymer interface, indicating a loss of adhesion. This behavior is consistent with the findings of Matos [26], who reported an increase in surface roughness in polylactic acid nanocomposites reinforced with cellulose microfibrils, attributing this effect to the formation of reinforcement agglomerates in the polymer matrix. Similar results were observed by Krapez et al. [36], who reported agglomeration of microcrystalline cellulose particles and the formation of voids or gaps between the polylactic acid matrix and the MCC particles, negatively affecting mechanical properties.

It was also found that SEM images of pure polylactic acid and composites with microcrystalline cellulose did not reveal the printing layers, which can be attributed to the 3D printing process using LCD (Liquid Crystal Display) with photopolymer resin. Unlike FDM (Fused Deposition Modeling) printing, which presents visible layers, the LCD method solidifies the resin layer by layer using UV light, resulting in a more homogeneous and cohesive surface. This absence of visible layers suggests good cohesion between the polymerized regions, which can positively influence mechanical properties, reducing failure points [10,42].

Therefore, the results of this study demonstrate that the incorporation of fillers into polylactic acid can modify fracture morphology. Small amounts maintain homogeneity, while high amounts favor aggregation and may impair interfacial adhesion.

### 3.7. Thermogravimetric Analysis

**Figure 18:**
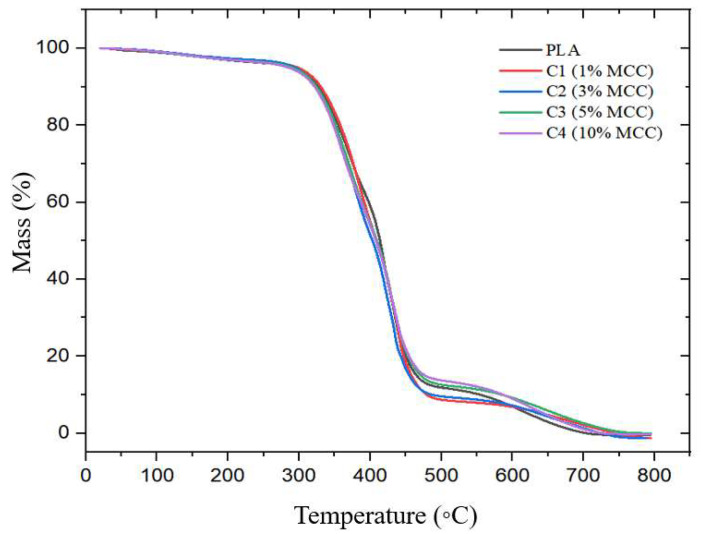
TGA curves for samples: C1 (1% MCC); C2 (3% MCC); C3 (5% MCC); C4 (10% MCC).

Figure 18 shows the thermogravimetric curves and the polymer composites (PLA + MCC) at different concentrations (1%, 3%, 5%, and 10% MCC). It is noted that, in all samples, there was only one degradation event, referring to the decomposition of the polymer chain, between the range of 250 and 500 °C, which was already expected, based on other works in the literature [43,44]. In the case of composites, from 300 °C onwards, it is attributed to the degradation of cellulose, the cellulosic material begins to degrade [24].

According to Figure 18 and Table 9, pure polylactic acid presented a mass loss with a Tonset of 255.54 °C. Composite C1 (1% MCC) presented a Tonset equal to that of pure polylactic acid. However, for samples C2 (3% MCC), C3 (5% MCC), and C4 (10% MCC), a reduction in Tonset was observed in relation to pure polylactic acid and sample C1, with values of 252.43 °C, 253.24 °C, and 253.48 °C, respectively. This decrease suggests that the addition of higher MCC contents may have impacted the thermal stability of the material.

**Figure 19:**
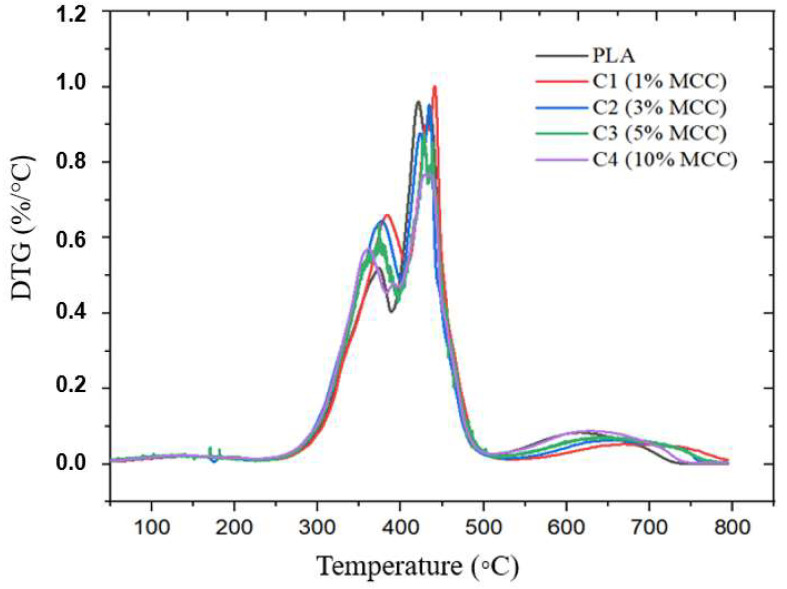
DTG curves for samples: C1 (1% MCC), C2 (3% MCC), C3 (5% MCC), and C4 (10% MCC).

In Figure 19, referring to the DTG analysis, a slight mass loss is observed in the analyzed samples. The observed values were 69.05% for pure PLA, 86.84% for C1 (1% MCC), 71.96% for C2 (3% MCC), 78.97% for C3 (5% MCC), and 79.16% for C4 (10% MCC). These results suggest that, while the addition of MCC can lead to variations in the thermal stability of the composites, in some cases, it also causes increased degradation, as observed in sample C1.

Contributing to the findings of this study, Matos [26] demonstrated in his research that there was a reduction in the degradation temperature when adding microfibrillated cellulose to polylactic acid, demonstrating the effect of cellulose on the thermal stability of the polymer. Sun [45] also observed that when adding coconut fiber to polylactic acid, the peak weight loss temperature of the composites shifted to the lower temperature region. Furthermore, it was found that the thermal stability of the composites decreased with increasing coconut fiber content, confirming the results found in this study on the influence of cellulose on the thermal degradation of PLA. Lee [1] also observed that the addition of nanocellulose to the polylactic acid matrix proportionally decreases the degradation onset temperature.

Therefore, the influence of MCC on the thermal degradation of PLA depends on its concentration, requiring strict control of the added content to balance thermal stability and biodegradation, especially in applications where material durability is critical.

### 3.8. Differential Scanning Calorimetry

**Figure 20:**
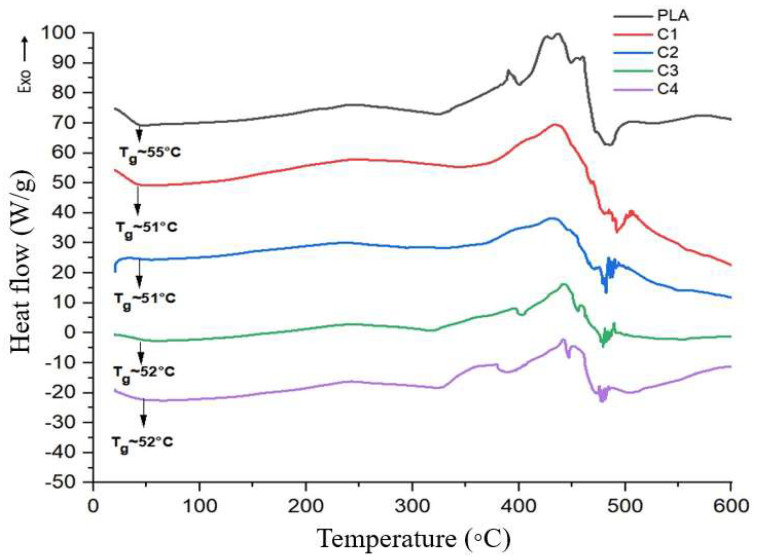
DSC curves for neat polylactic acid and composites (PLA + MCC).

Figure 20 shows the Differential Scanning Calorimetry (DSC) curves of pure polylactic acid and the composites containing microcrystalline cellulose (MCC). The glass transition temperature (Tg) was identified in each curve, and the values are summarized in Table 10. The Tg of pure polylactic acid was approximately 55 °C, a value consistent with those found by Silva [25] and Dias [42]. With the addition of microcrystalline cellulose (MCC), a slight reduction in the Tg of the composite samples was observed. This transition is an important parameter in thermal characterization, as it marks the onset of polymer chain mobility in the amorphous phase and identifies the limiting temperature for use of the amorphous polymer. This directly affects how the material behaves at different temperatures. Therefore, the decrease in Tg suggests that the presence of MCC may have influenced this mobility, possibly acting as a spacing or disorganization agent in the matrix, favoring the movement of the polymer chains.

Similar results were observed by Jesus [33], who found a Tg of around 53.6 °C when producing polylactic acid composites reinforced with microfibrillated cellulose. Campos [8] also obtained similar values, around 58 °C, when using cellulose extracted from pineapple peel as reinforcement in the PLA. It was also observed that the Tg showed a slight decrease as the percentage of cellulose increased. Sun [45] also found that with the incorporation of coconut fiber into the polylactic acid matrix, the Tg decreased, which indicates greater mobility and free volume of the matrix chains due to loose packing of charge within the matrix due to the poor interaction between the fiber and the PLA matrix. These results corroborate those obtained in this research.

### 3.9. Determination of Water Absorption of Polylactic Acid and Composites

**Figure 21:**
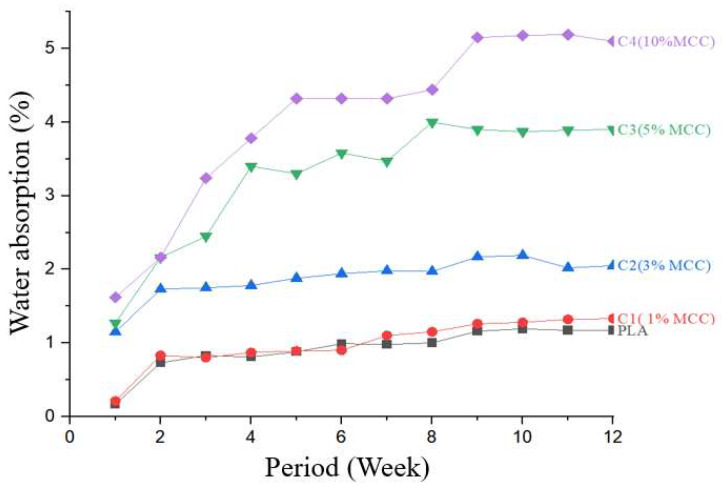
Water absorption curve for pure polylactic acid and composites (PLA + MCC) at different concentrations.

The water absorption test was performed on pure polylactic acid and composites; the absorption percentages are shown in Figure 21. From the analysis of the graph, it was found that polylactic acid presented the lowest water absorption values among the analyzed samples, registering only 1.17% at the end of 12 weeks. This behavior can be explained by the structure of PLA, which is a hydrophobic polymer, that is, it has low affinity for water. The absence of MCC in its composition contributes to this property, since MCC has a hydrophilic nature, in addition to there being no stress concentrators that could facilitate water penetration into the polymer matrix [24,40].

However, the composites presented higher water absorption values. Composite C4 (10% MCC) showed the highest index, reaching 5.10%, followed by the composite with 5% MCC, which presented approximately 3.90%. Sample C2 (3% MCC) presented 2.05% absorption. Sample C1 (1% MCC) registered an absorption of 1.33%. This progressive increase in water absorption with increasing MCC concentration can be attributed to the hydrophilic character of microcrystalline cellulose. MCC, being composed of natural lignocellulosic fibers, contains hydroxyl functional groups (-OH) in its structure, which interact with water molecules through hydrogen bonds [42]. Furthermore, the presence of microcrystalline cellulose causes a disorder in the polylactic acid macromolecules, creating less dense areas and increasing the material’s permeability to water. These disordered regions can act as pathways for water diffusion within the composite, contributing to the higher absorption rates observed [33].

These results are consistent with studies by Santos [40], who, when comparing pure polylactic acid with biocomposites containing natural fibers, observed that pure polylactic acid presented lower water absorption rates. This behavior is expected, since natural fillers, such as lignocellulosic fibers, have an affinity for water due to the presence of the aforementioned hydroxyl groups. These groups are primarily responsible for the fibers’ interaction with water, forming hydrogen bonds that facilitate absorption.

Similarly, Silva [24] found that composites with a higher fiber content presented greater water absorption. This behavior was explained by the hydrophilic nature of the natural fibers used, which have a greater capacity for interaction with water compared to pure polylactic acid. Furthermore, Silva highlighted that increasing fiber content tends to cause changes in the microstructure of the polymer matrix, creating microchannels or disordered regions that facilitate water diffusion within the material.

Therefore, this study confirms that incorporating microcrystalline cellulose into polylactic acid composites offers an opportunity to develop more sustainable materials, but highlights the need for strategies to reduce the effects of water absorption, such as surface treatments or chemical modifications, to improve performance in humid conditions.

## 4. Conclusions

The results obtained showed that the addition of microcrystalline cellulose to polylactic acid positively contributes to the mechanical performance of the composites, especially at a concentration of 3.00% (sample C2), which presented the best results in terms of tensile strength and modulus of elasticity. Shore D hardness analysis also indicated a significant increase with increasing MCC concentration. SEM morphological analysis demonstrated good dispersion of MCC in the PLA matrix at lower concentrations (1% and 3%), while higher concentrations (5% and 10%) presented heterogeneities that can negatively impact material performance.

From a thermal perspective, microcrystalline cellulose caused changes in thermal stability and, in some cases, favored thermal degradation, as observed in the sample with 1% MCC. After thermal aging, all samples showed a reduction in mechanical properties, demonstrating that the thermal degradation process compromises the integrity of the composites. XRD analysis showed increased crystallinity with the addition of MCC, while infrared spectroscopy (FTIR) did not indicate significant changes in the chemical structure of PLA. The increased water absorption with the higher MCC concentration was associated with its hydrophilic nature, a critical factor for applications in humid environments.

The application of photopolymerization 3D printing technology proved to be viable and innovative for the manufacture of PLA/MCC composites, representing a promising alternative for the production of functional, sustainable materials with potential applications in various fields, such as the automotive, biomedical, and environmentally friendly product industries.

It is therefore concluded that the incorporation of microcrystalline cellulose into PLA is an effective strategy for improving its mechanical properties, especially at moderate concentrations such as 3%. The combination with Additive Manufacturing can significantly expand the scope of applications for this biopolymer, promoting advances in the development of more sustainable, lightweight, and high-performance materials.

## Figures and Tables

**Table 1:** Parameters of impression.

Parameters	Values
Layer height (mm)	0.05
Exposure time (s)	2.5
Number of litters	5
Exposure time of base litter (s)	35
Lifting distance (mm)	5
Lifting speed (mm)	80
Retraction speed (mm)	210
Estimated printing time	10 min, 53 s

**Table 2:** Operating conditions used for XRD measurements for MCC.

Parameters	Values
Radiation	Co (*λ*_1_ = 1.789 Å)
Filter	Fe
Tube Voltage	40 K
Tube current	40 mA
Mask	10 mm
Anti-scatter slit incident	1/2°
Divergent slit	1/4°
Diffracted anti-scatter slit	7.5 mm
Detector	PIXcel
Step size	0.02
Time per step	200 s
Scan range	5°–40°

**Table 3:** Operating conditions used for DRX measurement.

Parameters	Values
Radiation	Co (*λ*_1_ = 1.789 Å)
Filter	Fe
Tube Voltage	40 K
Tube current	40 mA
Mask	10 mm
Anti-scatter slit incident	1/2°
Divergent slit	1/4°
Diffracted anti-scatter slit	7.5 mm
Detector	PIXcel
Step size	0.02
Time per step	200 s
Scan range	5°–40°

**Table 4:** FTIR band assignment of pure polylactic acid and PLA/MCC composites with identification of their respective functional groups.

Wavenumber (cm^−1^)	Assignment	References
812	C=O bond deformation (PLA crystalline phase)	Bartolomei (2016) [23]
987	C-COO stretching (PLA amorphous phase)	Bartolomei (2016) [23]
1108	Symmetrical stretching of the C-O-C group	Da Silva (2019) [24]
1196	Symmetrical stretching of the C-O-C group/C=O stretching	Da Silva (2019) [24]
1410, 1513, 1645	CH(CH3) vibration	Da Silva (2019) [24]
1715	C=O ester group stretching (methacrylate group)	Bartolomei (2016) [23]
2852	C-CH3 group stretching (PLA structure)	Da Silva (2019) [24]

**Table 5:** Tensile strength in MPa applied in the tensile test of polylactic acid and composites (PLA + MCC).

Tensile Strength in (MPa)			
Sample	Mean	Median	Standard Deviation
Pure PLA	19.50	20.25	1.84
C1 (1% MCC)	20.34	20.35	1.10
C2 (3% MCC)	24.15	23.98	2.08
C3 (5% MCC)	21.71	22.24	3.38
C4 (10% MCC)	20.30	19.75	1.50

**Table 6:** Modulus of elasticity in Giga Pascal (GPa) obtained in the tensile test of pure PLA and composites (PLA + MCC).

Sample	Mean ± S.D.	Median
Pure PLA	2.18 ± 0.14	2.21
C1 (1% MCC)	2.33 ± 0.14	2.14
C2 (3% MCC)	2.88 ± 0.18	2.81
C3 (5% MCC)	2.79 ± 0,24	2.82
C4 (10% MCC)	3.02 ± 0.23	2.93

**Table 7:** Shore D hardness of pure PLA samples and composites (PLA + MCC).

Sample	Mean ± S.D.	Median
Pure PLA	73.72 ± 0.30	74.50
C1 (1% MCC)	78.70 ± 0.55	78.70
C2 (3% MCC)	79.17 ± 0.27	70.16
C3 (5% MCC)	81.58 ± 0.88	81.58
C4 (10% MCC)	85.20 ± 0.23	85.20

**Table 8:** Crystallinity Index of crystalline microcellulose.

Sample	Total Area (u.a)	Area the Crystalline Region (u.a)		Crystallinity Index (%)
MCC	137,975.2877	Peak 1	22,651.21465	40.54
		Peak 2	25,924.43654	
		Peak 3	7361.273009	
		Total	55,936.92419	

**Table 9:** Thermal behavior of samples.

Thermal Decomposition Temperature			
Samples	T_onset_ (°C)	T_endset_ (°C)	Mass Loss (%)
Pure PLA	255.54	500.00	69.05
C1 (1% MCC)	255.54	511.61	86.84
C2 (3% MCC)	252.43	510.16	71.96
C3 (5% MCC)	253.24	515.01	78.97
C4 (10% MCC)	253.48	502.32	79.16

**Table 10:** Glass transition temperature (Tg) for neat PLA and composites.

Sample	Tg (°C)
Pure PLA	55
C1 (1% MCC)	51
C2 (3% MCC)	51
C3 (5% MCC)	52
C4 (10% MCC)	52

## Data Availability

The original contributions presented in this study are included in the article. Further inquiries can be directed to the corresponding authors.
